# New Appliances and Things Medical

**Published:** 1901-06-01

**Authors:** 


### NEW APPLIANCES AND THINGS MEDICAL.

[We shall be glad to receive, at our Office, 28 & 29 Southampton Street, Strand, London, W.C., from the manufacturers, specimens of all new preparations and appliances which may be brought out from time to time.]

### INVALID STOUT.

(BEAMISH & CRAWFORD, LIMITED, CORK. Wholesale Agents: GENDALL & CO., 34 Norfolk Street, Strand, London, W.C.).

THIS special stout—samples of which have been forwarded to us for examination—appears to be a liquor of first-rate quality. In addition to an excellent alcoholic percentage it contains those nutritive qualities which renders this form of malted beverage peculiarly valuable for invalids who require in addition to stimulating substances some easily-assimilated nutritive material.

### "VAMPIRE" FLY-CATCHER.

(KAY BROTHERS, LIMITED, STOCKPORT.)

The "Vampire" fly-catcher is a decidedly convenient form of trap and an improvement on the ordinary fly-papers. It consists of a spiral wire coated with an adhesive gum. The wire when pulled out to its full length is about three feet long. Each catcher is closely coiled up in an hermetically closed cartridge case; when wanted for use the free end of the wire, which projects outside the case, is pulled out, and the wire hung up in a suitable position. According as the Vampire is wanted for temperate or tropical climates, the gum is of corresponding degrees of consistence, the melting point being adapted to the temperature of the surroundings. The price of each catcher is only one penny. They will keep more or less indefinitely as long as the cartridge is not opened. They are easily stored and occupy little space, and they are less offensive to the eye than the ordinary fly-papers.

### GERM-FREE MILK.

(CRYSTAL BROOK FARM, THEYDON BOIS, ESSEX.)

THE germ-free milk, which is supplied from the Crystal Brook Farm, consists of two varieties, namely—the motherized and the invalid—both milks are stated to be the products of cows which have been submitted to the tuberculin test and found free from tuberculous disease, every possible precaution having been taken to ensure sanitary conditions during the handling of the milk. The milk is sterilised and bottled immediately after milking. The cows on the Crystal Brook Farm are said to be a Jersey herd, giving a very rich percentage of fat in the milk. Although we have no means of absolutely verifying these statements, an examination of the milk shows that it is a very clean milk and free from living germs, and thoroughly suited for the purposes for which it is intended. The motherized milk should be particularly valuable if the instructions which are supplied are accurately followed by those who employ the milk. The chief secret in rearing an infant on cows' milk is to give the proper quantities from the first feeding onwards. Mistakes made during the first few weeks of life are always difficult, and sometimes impossible, to rectify—the directions given with this germ-free milk are irreproachable. We would, however, point out that an infant fed indefinitely on sterilised milk may in some cases show signs of failure of nutrition owing to the want of some fresh element of food.

